# A task-specific interactive game-based virtual reality rehabilitation system for patients with stroke: a usability test and two clinical experiments

**DOI:** 10.1186/1743-0003-11-32

**Published:** 2014-03-06

**Authors:** Joon-Ho Shin, Hokyoung Ryu, Seong Ho Jang

**Affiliations:** 1 National Rehabilitation Center, Samgaksan-ro 58, Gangbuk-gu, Seoul, Korea; 2 Graduate School of Technology & Innovation Management, Hanyang University, 222 Wangsimni-ro, Seoongdong-gu, Seoul, Korea; 3 Department of Physical Medicine and Rehabilitation, College of Medicine, Hanyang University, 222 Wangsimni-ro, Seoongdong-gu, Seoul, Korea

**Keywords:** Virtual reality, Rehabilitation, Stroke, Paresis, Upper extremity, Video games

## Abstract

**Background:**

Virtual reality (VR) is not commonly used in clinical rehabilitation, and commercial VR gaming systems may have mixed effects in patients with stroke. Therefore, we developed RehabMaster™, a task-specific interactive game-based VR system for post-stroke rehabilitation of the upper extremities, and assessed its usability and clinical efficacy.

**Methods:**

A participatory design and usability tests were carried out for development of RehabMaster with representative user groups. Two clinical trials were then performed. The first was an observational study in which seven patients with chronic stroke received 30 minutes of RehabMaster intervention per day for two weeks. The second was a randomised controlled trial of 16 patients with acute or subacute stroke who received 10 sessions of conventional occupational therapy only (OT-only group) or conventional occupational therapy plus 20 minutes of RehabMaster intervention (RehabMaster + OT group). The Fugl-Meyer Assessment score (FMA), modified Barthel Index (MBI), adverse effects, and drop-out rate were recorded.

**Results:**

The requirements of a VR system for stroke rehabilitation were established and incorporated into RehabMaster. The reported advantages from the usability tests were improved attention, the immersive flow experience, and individualised intervention. The first clinical trial showed that the RehabMaster intervention improved the FMA (*P* = .03) and MBI (*P* = .04) across evaluation times. The second trial revealed that the addition of RehabMaster intervention tended to enhance the improvement in the FMA (*P* = .07) but did not affect the improvement in the MBI. One patient with chronic stroke left the trial, and no adverse effects were reported.

**Conclusions:**

The RehabMaster is a feasible and safe VR system for enhancing upper extremity function in patients with stroke.

## Background

Upper extremity (UE) functional deficits after stroke have received a great deal of attention because they are strongly related to the quality of life of stroke survivors [1,2]. Such deficits occur in approximately 70% of patients in the acute phase and persist in about half of patients in the chronic phase of stroke [3,4]. Hence, a variety of interventions have been suggested to improve UE function, of which high-intensity repetitive task-specific training appears to confer the greatest benefits [5].

However, it remains challenging to implement high-intensity repetitive training in the real clinical setting because the necessary resources may be limited [6]. In addition, many patients with stroke quickly lose interest in repetition-based training. For these reasons, virtual reality (VR)-based rehabilitation programs have gained medical attention as a novel therapeutic alternative for motor recovery after stroke.

VR is a computer-generated interactive simulation that imitates reality and provides users with an artificial environment including sensory information similar to real-world experience. It began to be employed specifically for rehabilitation about 15 years ago [7]. Previous studies have proven that VR can improve motor function in patients with stroke. Preliminary results in three patients with chronic stroke showed that use of a VR system improved range of motion, strength, and hand velocity [8]. In a trial by Piron et al., [9] 36 patients with stroke were randomly assigned to undergo VR-based rehabilitation or traditional physical therapy, and the VR-based group exhibited greater improvement in motor performance.

Recently, VR-based off-the-shelf commercial gaming systems, e.g. the Nintendo® Wii and Playstation EyeToy, have exhibited general physical effects when used in UE rehabilitation of stroke patients [10-12]. Some studies have examined the effects of similar systems in the field of rehabilitation. Chang et al. [13] showed that a Kinect^TM^-based system could be used as a rehabilitation tool in children with cerebral palsy and acquired muscle atrophy, and Ustinova et al. [14] demonstrated that a custom-made three-dimensional (3D) videogame enhanced arm postural coordination in patients with traumatic brain injury. However, these studies did not assess the functional outcomes and evaluate the effectiveness of the VR-based intervention in a randomised controlled trial. Notably, systems that were not originally developed for people with disabilities may produce mixed effects in some respects in patients with stroke [15]. To address these issues, we developed a VR-based rehabilitation system specifically for patients with stroke. In addition, we applied two principles of game design that are highly relevant to rehabilitation: *meaningful play*[16] and *challenges for scaffolding skill improvement*[17,18]. The aim of this study was twofold: 1) to develop a task-specific interactive game-based VR rehabilitation system for patients with stroke and 2) to assess its usability and clinical efficacy for UE rehabilitation of such patients.

## Methods

### Task-specific interactive game-based VR rehabilitation system

**Figure 1:**
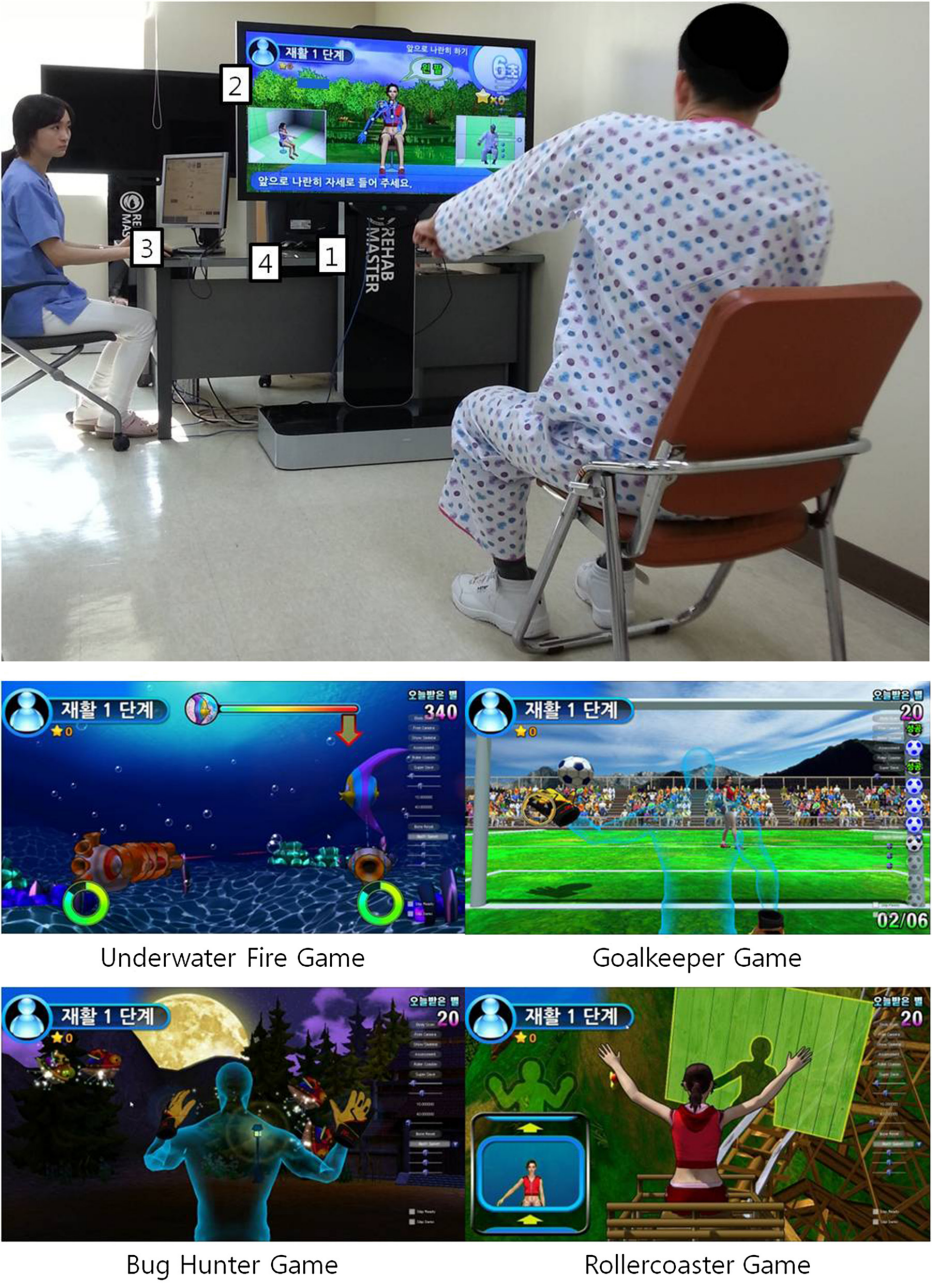
**View of the experimental setup of the RehabMaster system and a screen shot of a RehabMaster game.** The participant sits up in front of the monitor on which the program is projected. The participant is instructed to move his or her upper extremity (ies) and trunk in order to play the game. The RehabMaster system consists of: 1) a depth sensor, 2) a monitor with a built-in computer, 3) a monitor for the therapist, and 4) the RehabMaster system control computer for the therapist.

We developed a task-specific game-based VR rehabilitation system, called the RehabMaster™, which provides a rich interactive rehabilitation setting; this system is depicted in Figure 1. The patient sits in a chair in front of a monitor, facing an OpneNI™-compliant depth sensor (PrimeSense™ 3D awareness sensor, infrared projectors combined with standard RGB and infrared CMOS image sensors). The sensor is a Universal Serial Bus plug-and-play device that translates the scene geometry into depth information. From the point at which it is located, the sensor has an effective angle of 70°, a distance range of 0.8–3.5 m, and a response time of 10 ms and generates images of the participant with a resolution of 640 × 480 at 30 frames per second. A computer operated by Window 7 with a 2.9-GHz quad-core CPU and 4 GB SDRAM renders the images onto a 60-inch monitor with a resolution of 1920 × 1080. The RehabMaster is operated by the occupational therapist’s computer via a local area network, providing control of the patient’s training modules and the level of difficulty.

The main user interface for the RehabMaster comprises four elements: a *user management* module that contains information about each participant (e.g., an abbreviated medical record, the history of the patient’s RehabMaster sessions, and the therapist’s notes on those sessions), an *assessment* module that tracks the patient’s rehabilitation progress, a *rehabilitation training* program that asks the patients to imitate some of the 40 different motions performed by an avatar, and *rehabilitation games* that provide an engaging form of rehabilitation exercise using gaming concepts.

In detail, the assessment consists of evaluations of range of motion and movement evaluation with reference to such commonly used instruments as the Fugl-Meyer Assessment, [19] Action Reach Arm Test, [20] and Motricity Index [21]. Rehabilitation training simulates arm and trunk movements designed to restore specific functional deficits. The participant is able to practice various movements by copying specific motions made by the RehabMaster avatar. The motions incorporated were suggested by physiatrists and occupational and physical therapists specialising in stroke rehabilitation and were sufficiently numerous to provide suitable programs for participants with various deficits. The motions were intended to promote incremental improvement in range of motion and endurance, strength, and deviation from synergistic motion patterns. The rehabilitation games were designed to combine a variety of rehabilitation exercises with gaming elements, thus making the otherwise monotonous practice more competitive, motivating, interesting and enjoyable. Four different types of games that address general UE functional deficits in patients were suggested: *Underwater fire*, *Goalkeeper*, *Bug hunter*, and *Rollercoaster* (Figure 1). *Underwater fire* was designed to train the patient’s forearm movement and eye-hand coordination. The patient is asked to use two weapons to target the fish on the display by performing elbow flexion/extension and shoulder internal/external rotation. Here, the number of fish on the display and their trajectories are controlled by the occupational therapists. The therapists can also select an individual weapon in order to force the patients to use only the affected UE intensively. The number of fish terminated constituted the measure of game performance, and the difficulty of the game was determined by the size of the fish and the speed with which they moved on the display. The *Goalkeeper* and *Bughunter* games were designed to train UE control, endurance, speed, accuracy, and range of motion. The patient controlled a goalkeeper’s (or hunter’s) hands on the display to catch a football (or bug). The speed, location, trajectory of the football, and pattern in which the bugs appeared could be controlled by the occupational therapist. Finally, the *Rollercoaster* game was designed to increase the control, speed, and accuracy of UE and trunk movements. The game consists of imitating the postures displayed by the system, which simulate those adopted during a rollercoaster ride. That is, the patient is instructed to position his or her arms and trunk as shown by the avatar. The difficulty of the game is defined by the difficulty level of the postures and the speed of the rollercoaster. The patient’s actual movements during the entire gaming session are recorded and played back at the end of the session in order to provide feedback.

Prior to each RehabMaster intervention session, a physiatrist outlined the customised training and gaming tasks, which were then further modified by the occupational therapists during the actual training sessions.

### Participatory design and usability test

Three representative user groups, i.e. stroke patients, occupational therapists, and physiatrists, were involved in designing the RehabMaster. Each stroke patient’s routine tasks and procedures were evaluated individually and focus group studies were held once a week for around half a year. Feedback and suggestions were categorised and incorporated into the development process.

A usability study was then carried out in the same types of representative users. The main purpose of the usability test was to assess the RehabMaster from the perspective of each stakeholder group. The patients with stroke performed 20-minute RehabMaster sessions at regular intervals twice a week for two weeks under the supervision of occupational therapists and physiatrists. All three representative user groups completed a self-report-style five-point Likert questionnaire at the end of the RehabMaster intervention. The questionnaire was different for each user group in order to accommodate their different concerns.

As patient’s engagement is a key benefit of the RehabMaster intervention, the primary user group, i.e. the patients with stroke, assessed the ability of the RehabMaster to provide strong motivation, enjoyment, and consequently, an optimal flow experience [22]. The secondary user groups (occupational therapists and physiatrists), however, separately rated the usability of the RehabMaster from the perspective of whether it meaningfully improved upper limb dysfunction and whether it was capable of providing appropriate levels of challenge for all of the diverse patients in the stroke group. Here, we report on only those components relevant to the game design for stroke rehabilitation as rated by each of the three separate user groups.

### Clinical experiments

Patients with hemiparetic upper limb dysfunction secondary to first-ever stroke were recruited from two rehabilitation hospitals and the neurorehabilitation unit of a university hospital. All of them exhibited mild-to-severe deficits of the paretic upper extremity (≥2 and ≤4 on the Medical Research Council Scale [23], and ≥2 and ≤5 on the Brunnstrom stage of motor recovery for the proximal part of the upper extremity [24]). The exclusion criteria were pre-existing arm impairment, any painful condition affecting the upper limbs, difficulty in sitting for at least 20 minutes, severe cognitive impairment (mini-mental state examination score less than 10 points), and severe aphasia. The exclusion criteria were kept to a minimum in order to evaluate the feasibility of use of the RehabMaster among a variety of patients. All of the patients provided written informed consent to participate, and written informed consent for the publication of his clinical image was obtained from the patient. The study was conducted in accordance with the Declaration of Helsinki and approved by the Institutional Review Board of Hanyang University. In addition, the individuals of the Figure 1 gave permission to publish their images.

**Figure 2:**
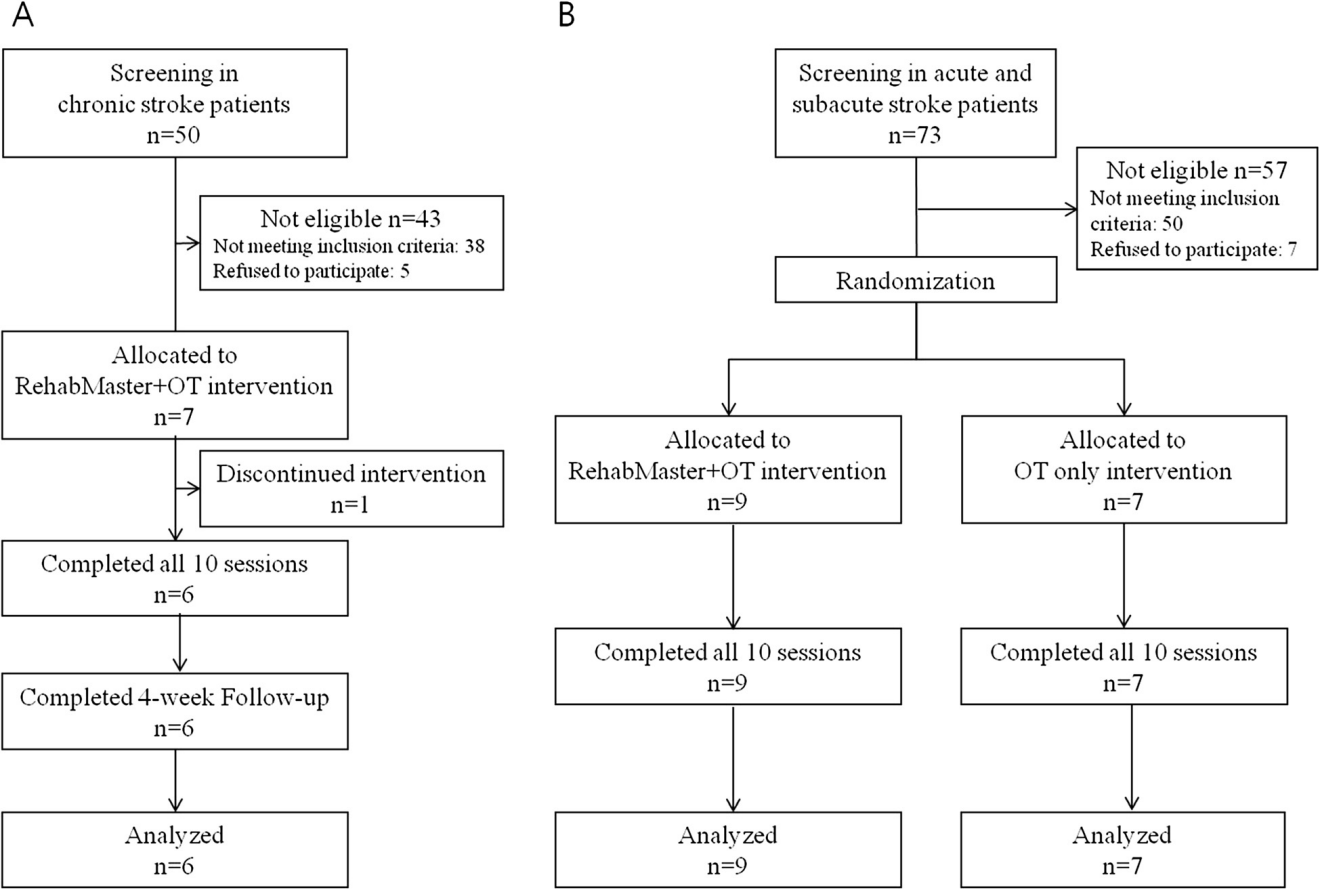
**Flowcharts of the clinical experiments. A.** Clinical experiments in patients with chronic stroke. **B.** Clinical experiments in patients with acute and subacute stroke.

Two consecutive clinical experiments were conducted. First, an observational study was performed in patients with chronic stroke in order to assess the feasibility of use and adverse effects of the RehabMaster-based training and games in patients with stroke (Figure 2A). All of the patients underwent UE rehabilitation consisting only of RehabMaster training. The patients performed 10 30-minute sessions (one session per day, five days per week for two weeks) for a total of 300 minutes of RehabMaster use. The Fugl-Meyer Assessment (FMA) for evaluation of upper limb motor function (0 = lowest score; 66 = highest score) [25] and the modified Barthel Index (MBI) (0 = lowest score; 100 = highest score) for global function evaluation [26] were administered at baseline (T0), during the fifth session (T5), during the last session (T10), and two weeks after intervention (T25) by independent evaluators blinded to the intervention. Adverse effects related to the RehabMaster intervention and the number of patients who dropped out of the study were also recorded.

Second, a prospective, single-blind, randomised controlled trial was conducted in patients with acute and subacute stroke (Figure 2B). The patients were randomly assigned to receive 10 sessions over two weeks of either conventional occupational therapy alone (OT-only group) or conventional OT plus 20 minutes of RehabMaster training (RehabMaster + OT group). The OT was delivered for 20 minutes by trained occupational therapists who were blinded to the protocol in order to provide participants the same OT used in the conventional clinical setting. The primary outcome was the FMA and the secondary outcomes were the MBI, Medical Research Council Score, and passive range of motion of the affected upper extremity. These assessments were made at baseline (T0) and during the last session (T10) by evaluators who were blinded to the type of intervention. Adverse effects related to the RehabMaster intervention and the number of patients who dropped out during the study period were also recorded.

### Statistical analysis

One-sample *t* tests against the neutral value in the five-point Likert rating were used to assess the responses to the six ‘*flow*’ statements [15]. A mean rating above 3.00 indicated that on average the patients agreed rather than disagreed with the statement [27]. To assess the flow experience provided by the RehabMaster, we examined four constructs shown by usability professionals to characterise the optimal flow state for learning activities: control, attentional focus, intrinsic interest, and curiosity [28]. Here, however, the last two constructs (i.e. intrinsic interest and curiosity) were combined as ‘Enjoyability’ for the patients.

Repeated measures one-way analysis of variance followed by post hoc tests was used to evaluate the effects of RehabMaster in patients with chronic stroke. In the patients with acute and subacute stroke, we conducted univariate analyses using Mann-Whitney tests to compare the changes in the FMA and MBI scores between the OT-only group and the RehabMaster + OT group. To verify the differences between the two groups, the baseline data were compared using the Mann-Whitney test and Fisher’s exact test. All analyses were performed using SPSS statistical software (version 17.0), and the level of statistical significance was *P* < .05 for all comparisons.

## Results

### Participatory design and usability test

We used several focus-group studies and interviews with the representative user groups (i.e. stroke patients, occupational therapists, and physiatrists) to establish the key design elements of an interactive VR rehabilitation system (Table 1). Those key elements were prioritised and incorporated into the RehabMaster.

**Table 1:** Key elements of interactive game-based virtual reality rehabilitation system

**Component**	**Key elements**
**Device**	Stable system, accuracy of the controller recognition
**Design**	Goal oriented task-specific contents, diversity of training and game contents not to lose interest, interactive and entertaining elements to be immersed in the game, tutorials to present explanation
**Difficulty**	Easy and slow to feel sense of accomplishment, adjustable to match individual level of performance and to maintain interest
**Scoring**	Scoring system to reflect exact performance status, Scoring to compete with other participants
**Sound**	Sound consistent with the results of performance for feedback, exciting and exaggerated effect sound to promote interest
**Graphics**	Simple graphics not to distract attention, Fun elements to provide positive experience

The advantages reported by each stakeholder group after initial testing of the RehabMaster can be summarised as follows: improved attention and an immersive flow experience for the patients with stroke; ability to follow the prescription and efficiently manage the intervention programs for the occupational therapists; and ability to administer effective individualised intervention for the physiatrists.

To see if RehabMaster afforded the stroke patients a desirable level of rehabilitation, we conducted a usability test in 20 patients with stroke and collected their responses as to whether they were highly engaged and considered the user experience pleasant, so that they were further motivated to take an active part in the RehabMaster intervention. Table 2 gives the scores across the three main components of the flow experience, i.e. attention maintenance, enjoyability, and motivation, which exhibited a consistent pattern. For all statements, the patients with stroke gave lower ratings for negative questions and higher ratings for positive questions. They found that the RehabMaster-based training and games maintained their attention strongly (statements 1 and 2) and were enjoyable (statements 3 and 4) without eliciting any negative feelings (statements 5 and 6).

**Table 2:** Ratings of the flow of the RehabMaster intervention by patients with stroke

**Statement**	**Rating**	**t**	**Significance**
1. I thought about other things when using RehabMaster (attentional focus)	0.8 ± 1.3	4.01	*P* < .01
2. I was aware of distractions when using RehabMaster (attentional focus)	0.6 ± 1.1	5.52	*P* < .01
3. Using RehabMaster was boring for me (intrinsic interest or pleasure)	0.5 ± 0.8	7.91	*P* < .01
4. RehabMaster was fun for me to use (intrinsic interest or pleasure)	4.3 ± 1.2	4.85	*P* < .01
5. I felt that I had control over my training process with RehabMaster (control)	4.1 ± 1.0	4.76	*P* < .01
6. I was frustrated with what I was doing when using RehabMaster (control)	0.9 ± 1.0	4.60	*P* < .01

The six ‘flow’ statements were adopted from [28]. All of the statements used in this study were deliberately rephrased in positive terms that the patients could easily understand.

To see if the challenges presented by the RehabMaster were of a level with which the patients with stroke could cope, we collected the field responses from three occupational therapists who employed the RehabMaster. All of them strongly agreed (5 out of 5) with both statements, ‘I was able to improvise the rehabilitation program using the RehabMaster in accordance with the actual performance of each patient’ and ‘I was easily able to manage the prescription using RehabMaster’. One of the occupational therapists stated that ‘Many patients were very satisfied with the adjustable difficulty of the rehabilitation program and were pleased to see that they were able to imitate the movements of the avatar on the screen correctly’.

To evaluate whether the game play constituted meaningful rehabilitation for the stroke patients, a semi-structured interview was administered to seven physiatrists who had employed the RehabMaster in patients with stroke. Most of the physiatrists strongly agreed that they had been able to design an effective rehabilitation program using the RehabMaster (six of seven physiatrists agreed that the RehabMaster seemed to be an effective method for administering OT) that could be tailored to the current state of each patient (all agreed that the RehabMaster was useful for customising the entire rehabilitation program for each patient, although one experienced physiatrist complained of its lack of specialised finger flexion and extension training). Further, all participating physiatrists felt that the RehabMaster was able to provide a record of relevant information concerning a patient’s rehabilitation progress. However, these statements were not amenable to any statistical evaluation because of the ceiling effect.

### Clinical experiments

Only one patient with chronic stroke discontinued the trial because of a personal issue unrelated to any adverse effect of the RehabMaster. None of the patients who participated in the RehabMaster intervention suffered from any adverse effect that would be likely to result from VR, such as dizziness or disorientation.

**Figure 3:**
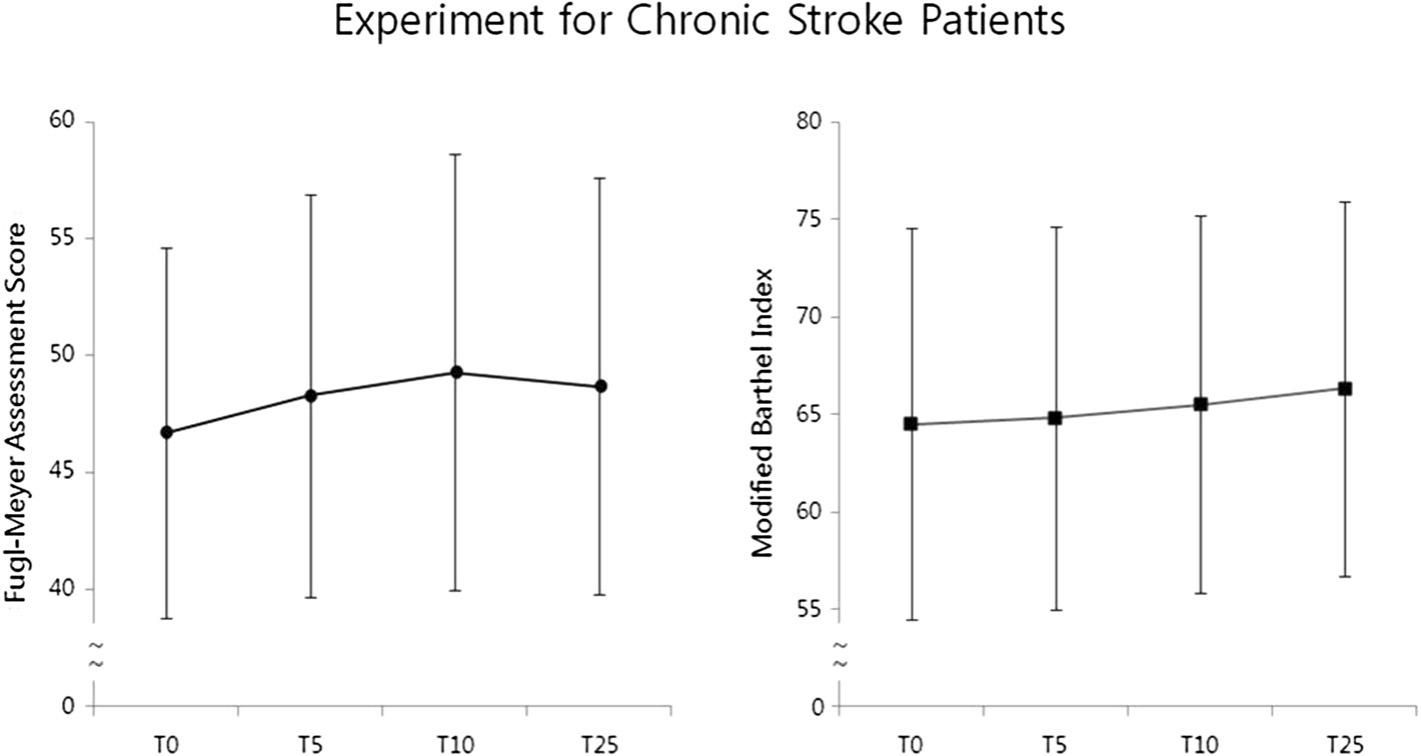
**Group mean change scores and standard error bars of Fugl-Meyer Assessment score of paretic upper limb and Modified Barthel Index in patients with chronic stroke.** Abbreviations: T0, baseline; T5, after the fifth session of intervention; T10, after tenth session of intervention; T25, two weeks after intervention.

In the first clinical experiment in patients with chronic stroke, six patients (six male patients, 48.7 ± 18.6 years old) completed two weeks of intervention and a follow-up evaluation during the fourth week. Figure 3 shows the results of the participants’ functional assessments at the four different time points. The results of repeated-measures ANOVA with a Greenhouse-Geisser correction are summarised in Table 3. Post-hoc tests using the Bonferroni correction indicated that the RehabMaster elicited slight but statistically insignificant improvements in the FMA score between T0 and T5 (*P* = .18) and between T5 and T10 (*P* = .25); although the FMA score then decreased by 0.67 between T10 to T25, this change was also not statistically significant (*P* = .61). Conversely, the MBI increased during all three intervals, T0 to T5 (*P* = .68), T5 to T10 (*P* = .68), and T10 to T25 (*P* = .44), indicating a steady and persistent effect over time.

**Table 3:** Results of repeated-measures ANOVA with a Greenhouse-Geisser correction on Fugl-Meyer assessment score of paretic upper limb and modified barthel index in patients with chronic stroke

**Source**	**df**	**Sum of squares**	**Mean square**	**F value**	**η**^**2**^	***P-*** **value**
FMA						
Time	1.335	23.167	17.348	7.092	0.586	0.029
Error	6.677	2.446				
MBI						
Time	1.470	11.792	8.020	5.145	0.507	0.047
Error	7.352	11.458	1.559			

*Abbreviations*: *FMA* Fugl-Meyer Assessment score, *MBI* modified Barthel index.

The second clinical experiment was performed in patients with acute or subacute stroke. None of the baseline characteristics differed significantly between the two groups (Table 4). The improvement in the FMA was greater in the RehabMaster + OT group than in the OT-only group, although this trend did not reach statistical significance (*P* = .07; Table 5). Although the improvement in the MBI did not differ significantly between the groups (*P* = .16; Table 5), the change in the MBI was greater in the RehabMaster + OT group (11.6 ± 6.5) than in the OT-only group (7.7 ± 4.6). The Medical Research Council Score and the painless passive range of motion of the affected upper extremity did not differ significantly between the two groups.

**Table 4:** Baseline characteristics of the experiments in patients with acute and subacute stroke

**Outcome**	**OT-only (n = 7)**	**OT + RehabMaster (n = 9)**	***P-*** **value**
Age, years	46.6 ± 5.8	52.0 ± 11.9	0.54
Male (%)	3 (42.9)	5 (55.6)	1.00^a^
Right-side lesion (%)	2 (28.6)	4 (44.4)	0.63^a^
Days after onset	76.6 ± 28.5	67.1 ± 45.3	0.30
mRS	3.7 ± 0.5	3.2 ± 1.0	0.40
FMA	34.4 ± 12.4	39.4 ± 10.7	0.46
MBI	44.7 ± 9.1	59.9 ± 17.6	0.10

*P-*value by Mann-Whitney test, ^a^*P-* value by Fisher’s exact test.

*Abbreviations*: *mRS* modified Rankin Scale, *FMA* Fugl-Meyer Assessment score, *MBI* modified Barthel index.

**Table 5:** Fugl-Meyer assessment score of paretic upper limb and modified barthel index in patients with acute and subacute stroke

**Time and group**		**FMA**	**MBI**
RehabMaster + OT	T0	39.4 ± 10.7	59.9 ± 17.6
	T10	51.1 ± 7.8	71.2 ± 15.4
OT-only	T0	34.4 ± 12.4	44.7 ± 9.1
	T10	40.7 ± 9.8	51.0 ± 8.8

*Abbreviations*: *FMA* Fugl-Meyer Assessment score, *MBI* modified Barthel index, *T0* before intervention, *T10* after tenth session of intervention.

## Discussion

The RehabMaster, a task-specific interactive game-based VR rehabilitation system, was developed to facilitate motor recovery after stroke. Our study included the first randomised controlled trial to assess the effects of a depth sensor-based VR gaming system on functional outcomes in patients with stroke; in addition, the testing of usability and clinical efficacy for upper extremity function in patients with stroke yielded favourable responses.

We employed a novel type of VR system, the RehabMaster, an OpenNI™-compliant depth sensor-based rehabilitation system that responds to the participant’s motions without the need for a controller or any attachments. This allows participants who have not regained sufficient hand power to use a game controller to interact with the system. In contrast, previous VR gaming systems have required the participants to be able to grasp a controller or to wear gloves and coloured patches on their upper extremities [11,29,30]. Our system can thus be utilised more extensively beginning in the initial phase of recovery and in patients with severe hemiplegia.

The FMA and MBI improved during the RehabMaster intervention in patients with chronic stroke. As none of the patients with chronic stroke in our clinical trials was receiving any other kind of therapy at the time of recruitment, these improvements appeared to indicate that the RehabMaster intervention was effective in patients with chronic stroke. In addition, the randomised controlled trial in patients with acute/subacute stroke also showed that RehabMaster + OT elicited greater improvement in FMA or MBI compared to OT-only groups, although this trend did not reach statistical significance. Therefore, RehabMaster might be a useful novel tool for rehabilitation of the upper extremities in patients with stroke. We speculated that these functional improvements stemmed from the greater focus of the RehabMaster intervention on the affected upper extremity [31]. As seen in the EXCITE trial (Extremity Constraint Induced Therapy Evaluation), the intensive use of the affected arm may contribute to successful rehabilitation, even in the chronic stage of stroke [32,33]. The task-specificity of the RehabMaster, which includes more than 40 kinds of training and games with different purposes, might also have been helpful in this regard. This property was created by the design of the RehabMaster specifically for patients with UE functional deficits due to stroke.

As the observation of action contributes to motor recovery by mirror motor neuron activation, [34] the patient’s viewing of the avatar’s movements on the screen may also have assisted the functional improvement The real-time natural interaction between the patient and the avatar on the screen that RehabMaster promotes might boost this action observation effect. On the other hand, the interactive nature of the system also increases the user’s awareness of his or her own movement. Functional improvement via rehabilitation in patients with stroke is best accomplished by providing an appropriate level of challenge for the patient’s current skill level and thus motivating the patient to engage. Meaningful play emerges from the relationship between the patient’s actions and the outcome on the system and also from the close relationships between the outcomes and the goals of the rehabilitation. The ability to adjust the level of difficulty gradually in accordance with the patient’s progress was a highly appreciated feature of the RehabMaster. Of course, this could also have been accomplished with a rule-based system or an artificial intelligence system to adjust the program in response to each patient’s individual level of performance. However, such a system would be difficult to achieve at this time given that a game designer cannot possibly know the current state of and best individual treatment protocol for every patient in advance. Instead, the RehabMaster allows occupational therapists, who are in direct contact with the patients, to make the desired adjustments. The practice data provided by the RehabMaster helped the therapists to devise new sets of individualised tasks for the patients to practice. Hence, as the rehabilitation continued over a period of weeks, the therapists could increase the level of difficulty of the intervention to ensure that the patients with stroke continued to be optimally challenged.

Moreover, the usability test indicated that the stroke patients received a ‘flow experience’. We suspect that this flow experience results from a combination of intrinsic motivation and complete immersion in the intervention [22]. This may also have helped to minimise the number of patients who dropped out of the experiments. This is unsurprising because RehabMaster was specifically designed to incorporate game elements faithfully and with consideration for the characteristics of patients with stroke.

Another main concern in the real-world rehabilitation setting is how to treat patients safely. This may be an important advantage of the RehabMaster when considered as a legitimate rehabilitation intervention to be adopted by a medical institution or medical insurance system. The complete absence of adverse effects during the intervention suggests that the RehabMaster is a safe rehabilitation tool. As the intervention is performed in the sitting position, there is a lower risk of falling, which is a common hazard in patients with stroke and the elderly. The supervision by occupational therapists also increases the level of safety.

Our study has several limitations that must be considered when interpreting the results. We first tested the RehabMaster in patients with chronic stroke, as most previous studies using VR were performed in such patients [35]. Once it was established that the system was safe in patients with chronic stroke, we enrolled patients with acute and subacute stroke in the second trial. Therefore, we intended to demonstrate the effects of the RehabMaster in a non-controlled clinical trial in patients with chronic stroke and in a randomised controlled trial in patients with acute/subacute stroke. The results from two trials, however, showed a slight difference. The different rehabilitation goals and characteristics of each phase of stroke might have influenced the results in these two groups. However, the present study was a pilot study originally designed to test the feasibility of using the RehabMaster in patients with varied degrees and stages of stroke. Different experimental protocols using different intervention times in the two experiments may have caused the inconsistency in their results. We attempted to determine the feasibility of using the RehabMaster for rehabilitation according to the benefit catalogue from the National Health Insurance Services of the Republic of Korea, which includes 20 and 30 minutes of OT. Therefore, both 20- and 30-minute RehabMaster sessions were employed, and the results imply that both durations of RehabMaster intervention are feasible for upper extremity rehabilitation. In the near future, an investigation focused on a specific population with a consistent protocol will be needed in order to establish an appropriate rehabilitation protocol. The differences between the groups of patients with acute/subacute stroke at baseline, despite their statistical non-significance, and the relatively short follow-up period were also limitations of the current study.

Another limitation is that the assessments in the clinical experiments were restricted to functional outcomes (FMA and MBI) and a few motor-related factors (range of motion and strength). The present study would have been strengthened by the use of measures based on the participants’ natural environments, which might have indicated whether the effects of VR rehabilitation are generalisable to the real world. In addition, we did not appraise other factors, such as cognitive function, motivation, and depression, which are commonly examined in patients with stroke. Finally, we evaluated the satisfaction or enjoyment in the usability test but did not compare it between the groups in the clinical trial.

Therefore, various aspects of the effects of the RehabMaster should be confirmed in future comparative studies with the comparison between groups receiving the same total amount of intervention time in order to eliminate any confounding by this factor. Finally, we plan in the future to evaluate the kinematic data recorded in real time during the RehabMaster intervention.

## Conclusions

The present study described the development of a task-specific, interactive, game-based VR rehabilitation system, called the RehabMaster**™**, and presented the results of a usability test and clinical trials. The RehabMaster proved to be a feasible and safe rehabilitation tool to enhance motor function among patients in various stages of recovery after stroke. It also encouraged the patient’s skill development, improved immersion, and motivated further rehabilitation by providing meaningful play, optimal challenge, and a flow experience.

## Abbreviations

UE: Upper extremity; VR: Virtual reality; FMA: Fugl-Meyer Assessment; MBI: Modified Barthel Index; OT: Occupational therapy; EXCITE: Extremity constraint induced therapy evaluation.

## Competing interests

The authors declare that they have no conflicts of interest with respect to the authorship and/or publication of this article.

## Authors’ contributions

JHS contributed to the conception and design of the RehabMaster, data acquisition, analysis and interpretation of data, and drafting of the manuscript. HR designed and analysed the usability test, provided technical and material support, and revised the present manuscript. SHJ designed the study, completed the statistical analysis, interpreted data, obtained funding, and critically reviewed the manuscript for important intellectual content. All authors read and approved the final manuscript.
